# Supplementary figures and images for: Development of Negative Controls for Fc-C-Type Lectin Receptor Probes
Source: Microbiol Spectr. 2023 May 9;11(3):e01135-23. doi: 10.1128/spectrum.01135-23 (PMC10269840; doi:10.1128/spectrum.01135-23)

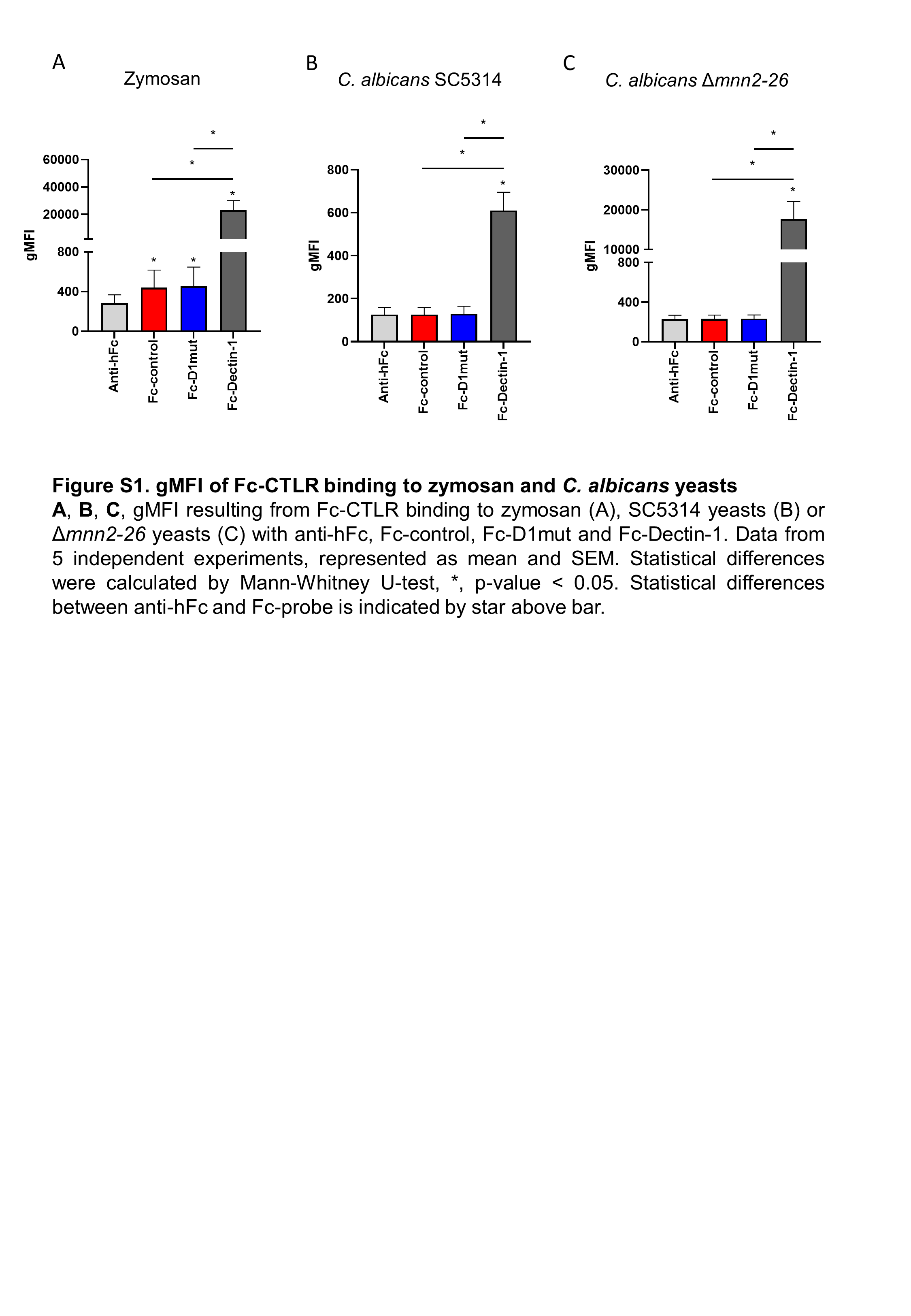

Supplement: Supplemental file 1 — Figure S1. Download spectrum.01135-23-s0001.tif, TIF file, 0.7 MB [file spectrum.01135-23-s0001.tif]

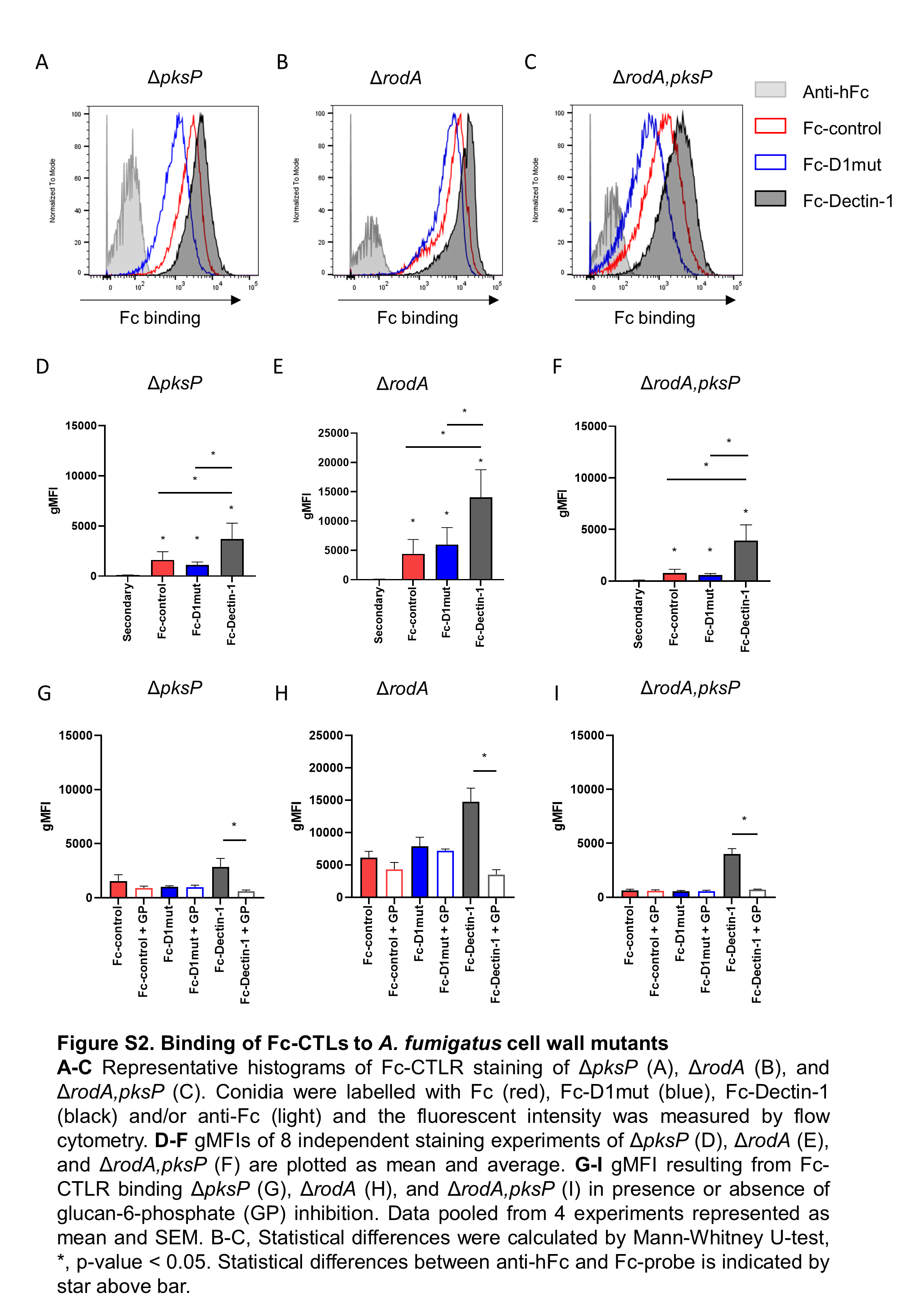

Supplement: Supplemental file 2 — Figure S2. Download spectrum.01135-23-s0002.tif, TIF file, 1.2 MB [file spectrum.01135-23-s0002.tif]

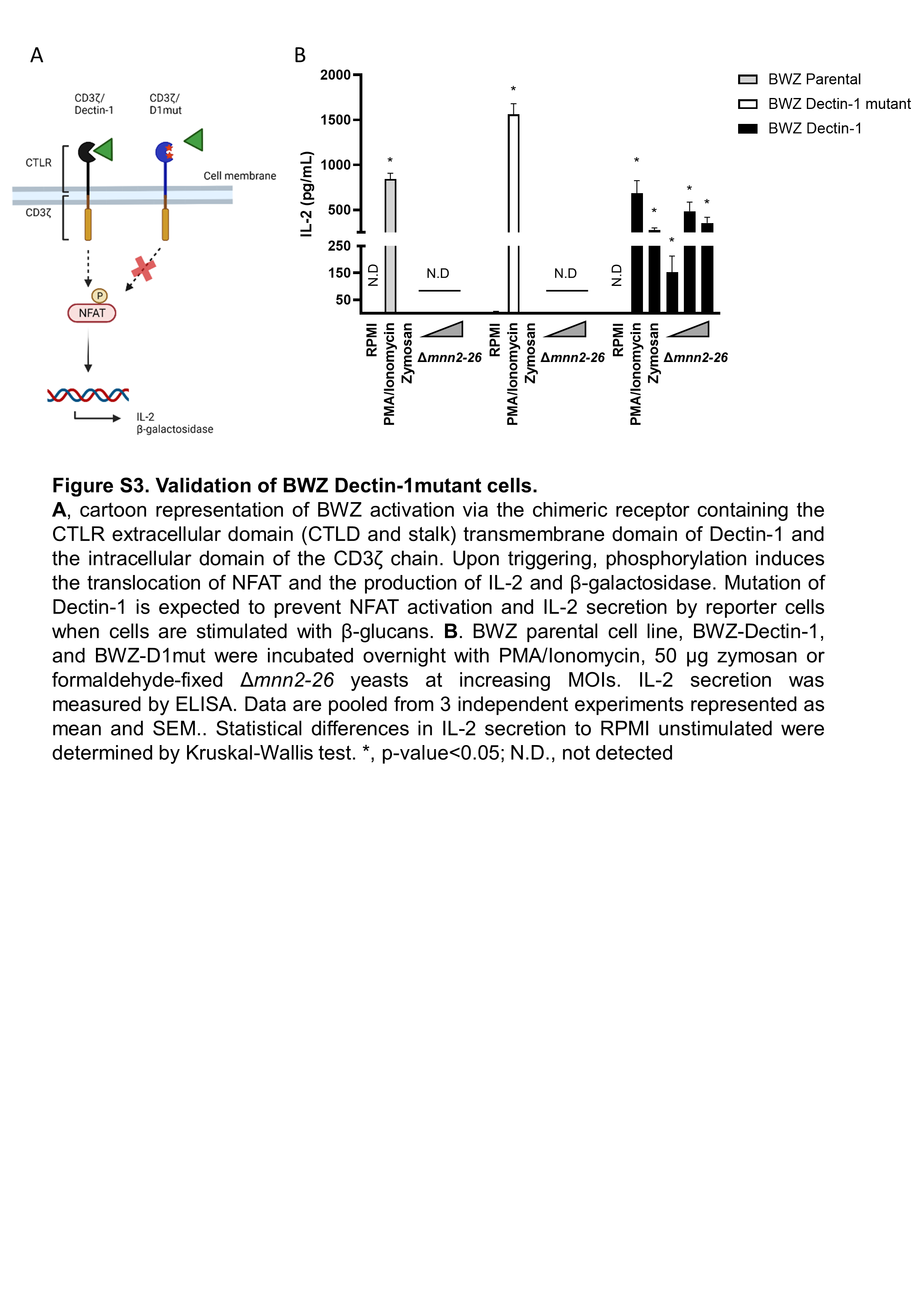

Supplement: Supplemental file 3 — Figure S3. Download spectrum.01135-23-s0003.tif, TIF file, 0.9 MB [file spectrum.01135-23-s0003.tif]
